# Inpatient multimodal occupational rehabilitation reduces sickness absence among individuals with musculoskeletal and common mental health disorders: a randomized clinical trial

**DOI:** 10.5271/sjweh.3882

**Published:** 2020-07-01

**Authors:** Sigmund Ø Gismervik, Lene Aasdahl, Ottar Vasseljen, Egil A Fors, Marit B Rise, Roar Johnsen, Karen Hara, Henrik B Jacobsen, Kristine Pape, Nils Fleten, Chris Jensen, Marius S Fimland

**Affiliations:** Department of Public Health and Nursing, Faculty of Medicine and Health Sciences, NTNU, Norwegian University of Science and Technology, Trondheim, Norway; Department of Physical Medicine and Rehabilitation, St. Olavs Hospital, Trondheim University Hospital, Trondheim, Norway; The Norwegian Labour and Welfare Service of Trøndelag, Trondheim, Norway; UnicareHelsefort Rehabilitation Center, Rissa, Norway; Department of Mental Health, Faculty of Medicine and Health Sciences, NTNU, Norwegian University of Science and Technology, Trondheim, Norway; Norwegian Advisory Unit on Complex Symptom Disorders, St. Olavs Hospital, Trondheim University Hospital, Trondheim, Norway; Department of Pain Management and Research, Oslo University Hospital, Oslo, Norway; Department of Community Medicine, UiT The Arctic University of Norway, Tromsø, Norway; National Center for Occupational Rehabilitation, Rauland, Norway; Department of Neuromedicine and Movement Science, Faculty of Medicine and Health Sciences, NTNU, Norwegian University of Science and Technology, Trondheim, Norway

**Keywords:** Key terms cognitive behavioral therapy, fatigue, health services research, inpatient care, musculoskeletal ­diseases, occupational rehabilitation, physical exercise, problem solving, psychiatry, return to work

## Abstract

**Objectives:**

This study aimed to investigate whether inpatient multimodal occupational rehabilitation (I-MORE) reduces sickness absence (SA) more than outpatient acceptance and commitment therapy (O-ACT) among individuals with musculoskeletal and mental health disorders.

**Methods:**

Individuals on sick leave (2-12 months) due to musculoskeletal or common mental health disorders were randomized to I-MORE (N=86) or O-ACT (N=80). I-MORE lasted 3.5 weeks in which participants stayed at the rehabilitation center. I-MORE included ACT, physical exercise, work-related problem solving and creating a return to work plan. O-ACT consisted mainly of 6 weekly 2.5 hour group-ACT sessions. We assessed the primary outcome cumulative SA within 6 and 12 months with national registry-data. Secondary outcomes were time to sustainable return to work and self-reported health outcomes assessed by questionnaires.

**Results:**

SA did not differ between the interventions at 6 months, but after one year individuals in I-MORE had 32 fewer SA days compared to O-ACT (median 85 [interquartile range 33–149] versus 117 [interquartile range 59–189)], P=0.034). The hazard ratio for sustainable return to work was 1.9 (95% confidence interval 1.2–3.0) in favor of I-MORE. There were no clinically meaningful between-group differences in self-reported health outcomes.

**Conclusions:**

Among individuals on long-term SA due to musculoskeletal and common mental health disorders, a 3.5-week I-MORE program reduced SA compared with 6 weekly sessions of O-ACT in the year after inclusion. Studies with longer follow-up and economic evaluations should be performed.

Musculoskeletal and common mental health disorders are the major causes of disability and working years lost in the western world (1-4). For musculoskeletal disorders, effective occupational rehabilitation programs have comprised multimodal interventions including components such as physical exercise, psychological/behavioral therapy, work-related problem solving and often involvement and coordination of different stakeholders (5, 6). For individuals with musculoskeletal or common mental health disorders, a recent meta-analysis concluded that psychological treatments reduce sick leave more than usual care, albeit with small effect sizes, and inconclusive results as to which form of psychological treatment is the most effective (7).

The worker`s decision to remain off or return to work involves complex interactions between personal beliefs, physical, psychosocial, and system factors and goes far beyond the medical treatment paradigm for any specific diagnosis (8, 9). In addition, co-morbidity between musculoskeletal pain and mental health disorders is high (10–12). Successful occupational interventions for individuals with musculoskeletal disorders have recently inspired the development of similar promising interventions for common mental health disorders (5, 13).

Acceptance and commitment therapy (ACT) is a recent development within cognitive behavior therapy with empirical support as a coping strategy for a broad range of clients (14), including for individuals with musculoskeletal and common mental health disorders (15–17). A Swedish randomized pilot study reported fewer sickness absence (SA) days in women with musculoskeletal complaints receiving ACT (18). Furthermore, ACT has successfully been implemented as a coping modality in group-based interventions for sick-listed individuals with different diagnoses (12, 19, 20).

We have previously compared a short (8 days) inpatient rehabilitation program to group-based outpatient ACT (O-ACT) for patients sick-listed due to musculoskeletal or common mental health disorders. We found no significant differences in SA between this short inpatient program and 6 weeks of O-ACT during one year of follow-up (21), and there were negligible differences in self-reported health outcomes (22). However, in Norway, 3–4 weeks of inpatient multimodal occupational rehabilitation (I-MORE) is common for individuals with complex biopsychosocial barriers for return to work. Effects of such programs have never been assessed in a rigorous design.

The aim of this study was to compare the effect on SA of 3.5 weeks I-MORE to the 6 weekly sessions of O-ACT. We hypothesized that the more comprehensive I-MORE would reduce SA compared to O-ACT.

## Method

The Regional Committee for Medical and Health Research Ethics in Central Norway approved this open label parallel randomized clinical trial (No.: 2012/1241), registered in clinicaltrials.gov (No.: NCT01926574), and adhered to the CONSORT statement (23). The study protocol is published elsewhere (24).

### Eligibility criteria

Participants aged 18–60 years sick-listed (2–12 months, current sick leave status ≥50%) due to a musculoskeletal, psychological, or general and unspecified disorder (eg, fatigue) as classified by ICPC-2 (the International Classification of Primary Care, second edition) were included. The exclusion criteria were: (i) alcohol or drug abuse; (ii) serious somatic disease (eg, cancer, unstable heart disease) or mental disorder (eg, high suicidal risk, psychosis, ongoing manic episode); (iii) disorders requiring specialized treatment; (iv) pregnancy; (v) current participation in another treatment or rehabilitation program; (vi) insufficient oral or written Norwegian language skills to participate; (vii) surgery scheduled within the next six months; and (viii) serious problems with functioning in a group setting, as assessed by a multidisciplinary team.

### Recruitment of participants

The Norwegian Labor and Welfare Administration identified and randomly invited potential participants from its records. Potential participants were asked to respond to the invitation either in writing or by telephone contact with a project co-worker. The project co-worker excluded individuals that self-reported any of the exclusion criteria. We invited the remaining candidates to outpatient assessment of eligibility consisting of individual appointments with a psychologist, a physiotherapist and a physician. This multidisciplinary team made a joint decision on whether the eligibility criteria were met.

### Randomization and blinding

Eligible participants were randomized to either I-MORE or O-ACT. The Unit of Applied Clinical Research (third party) at the Norwegian University of Science and Technology (NTNU) conducted the randomization by a flexibly weighted procedure, which ensured that the rehabilitation center had enough participants to run monthly groups in periods of low recruitment. One of the researchers analyzed the primary outcomes while blinded to allocation. It was not feasible to blind primary researchers in preparation and analysis of the dataset due to knowledge of the unequal group sizes.

### Interventions

The I-MORE program was provided at Hysnes rehabilitation center located in a rural setting one-hour travel from St. Olavs hospital in the city of Trondheim, Norway. I-MORE lasted 3.5 weeks and was more comprehensive than O-ACT, which mainly consisted of group-based ACT (2.5 hours/week for 6 weeks) at St. Olavs hospital. The length of the inpatient and outpatient interventions reflected common clinical practice. I-MORE comprised various treatment modalities such as physical exercise, work-related problem solving and a development of a written return-to-work plan in addition to ACT, whereas O-ACT consisted mainly of ACT. Mindfulness was integrated in several elements within both interventions. Details of the two programs are described in table 1 and in the protocol article (24). Adherence to- and competence in ACT was ensured by the same peer reviewed ACT trainer through video supervision and mentoring of the clinicians in both interventions.

**Table 1:** Overview of the rehabilitation programs ^a^ [ACT= acceptance and commitment therapy; GP=general practitioner.]

	Inpatient multimodal occupational rehabilitation (I-MORE)	Outpatient acceptance and commitment therapy (O-ACT)
Location	Inpatient rehabilitation center	Outpatient Hospital clinic
Duration	3.5 weeks (supervised sessions: 45.5 hours)	6–7 weeks (supervised sessions: 18.5 hours)
Contents and qualities	- group discussions (×8, total 16 hours; ACT based) - psychoeducational sessions (×4, total 6.5 hours) - individual meetings with coordinator (×5, total 5 hours) - individual meeting with physician (×1, 0.5 hours) - supervised physical exercise sessions (×10, total 12 hours) - outdoor activities day (×1, 5 hours) - “network day” with 2 group sessions (total 4 hours) - mindfulness sessions (×7, total 3.5 hours) ^b^ - “walking to work” (×6, total 3 hours) ^b^ - create return to work plan - at least one weekend at home framed as “home practice” ^b^ - a resume of the return to work plan was sent to the GP	- weekly ACT group sessions of 2.5 hours duration (×6, total 15 hours led by physician or psychologist) - group discussion on physical activity (×1, 1 hour led by a physiotherapist) - individual sessions (×2, total 2 hours with social worker trained in ACT) - individual closing therapy session in week 6 or 7 with both the social worker and the group therapist present (×1, 0.5 hours) - 15 minutes mindfulness at the start of group sessions (× 6, total 1.5 hours) - home practice, including daily mindfulness (15 minutes audio guided) ^b^ - a short resume of the program content and the patient’s own value based action plan was sent to the GP after the individual closing session.

a Adapted from protocol article; Fimland et al. BMC Public Health 2014.

b Scheduled but not supervised parts of the program.

### Outcome measures

The primary outcome measures were the cumulative number of SA days (total number of whole workdays lost) within 6 and 12 months follow-up (see statistics section for details). Secondarily, time until sustainable return-to-work (4 weeks without SA) was assessed up to 12 months. The SA data are based on medically certified SA, work assessment allowance and changes in permanent disability pension during follow up, obtained from the National Social Security Registry. Employees at the Norwegian Labor and Welfare Service registered and provided SA data. They were blinded to treatment allocation.

Self-reported secondary health outcomes were pain (25), anxiety and depression symptoms (26), subjective health complaints (27) and health-related quality of life (28), all measured as continuous scale scores and described in detail previously (21, 22). The participants answered web-based questionnaires at baseline, at the start and the end of the interventions, and at 3, 6 and 12 months of follow-up.

### Sample size

The sample size calculations are described in detail elsewhere (21, 29). An average SA of 60 [standard deviation (SD) 40] and 90 (SD 60) days for I-MORE and O-ACT respectively, would require 61 persons for each group. We aimed to include 80 persons in each arm allowing for 20% attrition or loss to follow-up.

### Statistical analysis

The cumulative number of SA days at 6 and 12 months after inclusion were calculated and compared for the two programs using the Mann-Whitney U-test (30). Sickness absence days were calculated according to a 5-day workweek adjusted on a monthly basis for part-time employment, partial sick leave and changes in permanent (partial) disability benefits, enabling a count of cumulative days compensated with benefits (total number of whole workdays lost) (21). We graphically displayed differences by plotting the median number of SA days in each intervention group as a function of time (cumulative median). For time until sustainable return to work, Kaplan Meier curves were estimated and compared using the log rank test (30). Return-to-work hazard ratios were estimated using the Cox proportional hazard model and the Efron method for ties (31), with and without adjustment for gender, age, education, main diagnosis for sick leave and length of sick leave at inclusion. Time was calculated as the number of months from inclusion, and participants were censored at the first month without SA or at the end of follow-up (12 months). The proportionality hazards assumption was tested using the Schoenfeld Residual test (32). Self-reported health outcomes were analyzed as repeated measurements over time using linear mixed models (33), modelled without random slope (only random intercept) if the full model did not converge. Analyses were performed according to the intention-to-treat principle. Additional per protocol analyses were done by excluding participants that withdrew after randomization (before or during the programs) and/or attended less than 60% of the sessions of O-ACT.

We performed sensitivity analyses with sustainable return to work defined as 2 and 3 months without receiving benefits. We considered P<0.05 (two-tailed) to be statistically significant. Precision of the estimates was assessed by 95% confidence intervals (CI). All analyses were done using STATA 13.1 (StataCorp, College Station, TX, USA).

## Results

**Figure 1:**
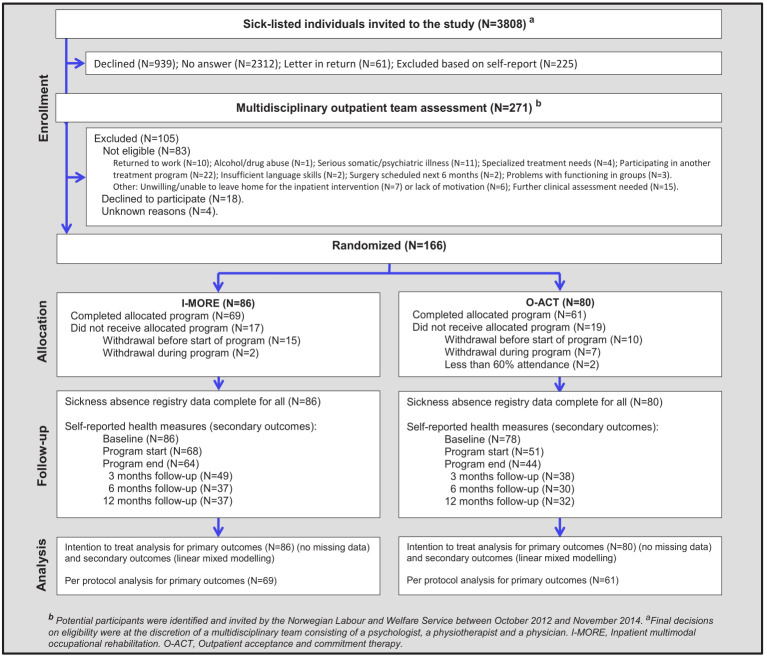
Flow of participants in the study.

Of 3808 persons invited to take part in the study, 271 accepted the invitation and 166 were randomized to I-MORE (n=86) or O-ACT (n=80). See figure 1 for information about the flow of participants, dropouts and missing data.

### Participants’ characteristics

The mean age of the participants was 46 (SD 9.5) years and the majority was women (79%). About 60% of the participants did not have education beyond high-school level, and the median length of sick-leave reimbursement during the last 12 calendar months prior to inclusion was 210 calendar days (IQR 170-265). Baseline characteristics for the two intervention groups showed only minor differences (table 2).

**Table 2:** Participants’ baseline characteristics. [HADS=hospital anxiety and depression scale ICPC2=international classification of primary care, 2^nd^ edition; I-MORE=inpatient multimodal occupational rehabilitation; IQR=interquartile range; NRS=numeric rating scale; O-ACT=outpatient acceptance and commitment therapy; SD=standard deviation.]

Variables	I-MORE (n = 86)		O-ACT (n = 80)	
	
	N (%)	Mean/median (SD/IQR)	N (%)	Mean/median (SD/IQR)
Age ^a^		46.3 (8.7)		45.2 (10.4)
Women ^a^	70 (81)		61 (76)	
Higher education (university/college) ^b^	32 (37)		34 (43)	
Work status ^a, b^				
No work	11 (13)		6 (8)	
Full time	54 (63)		53 (66)	
Part time	21 (24)		21 (26)	
Graded disability pension ^c^	9 (10)		6 (8)	
Sick leave status at inclusion ^a^				
Full sickness benefit	35 (41)		36 (45)	
Partial sickness benefit	48 (56)		38 (48)	
Work assessment allowance ^d^	3 (3)		6 (8)	
Length of sick leave at inclusion ^a, e^		204 (163–265)		216 (177–265)
Sick leave diagnoses (ICPC-2) ^a^				
Musculoskeletal diagnosis	54 (63)		40 (50)	
Psychological diagnosis ^f^	32 (37)		40 (50)	
Anxiety HADS score (0–21) ^b^		7.4 (3.9)		8.6 (4.1)
Depression HADS score (0–21) ^b^		5.7 (4.2)		6.6 (4.0)
Average pain NRS (0–10) last week ^b^		5.0 (2.0)		4.8 (2.2)
Strongest pain NRS (0–10) last week ^b^		6.5 (1.9)		6.2 (2.5)

a Based on registry data.

b Based on self-reported data.

c Individuals working ≥50% at inclusion alongside graded permanent disability pension.

d Work assessment allowance is a medical benefit usually received after reaching the maximum of one year on sick leave benefits in Norway.

e Number of days on sick leave during the last 12 months prior to inclusion. Measured as calendar days, not adjusted for partial sick leave.

f Four I-MORE and nine O-ACT participants with fatigue and one I-MORE participant with perinatal distress included here.

### Sickness absence and return to work

**Figure 2:**
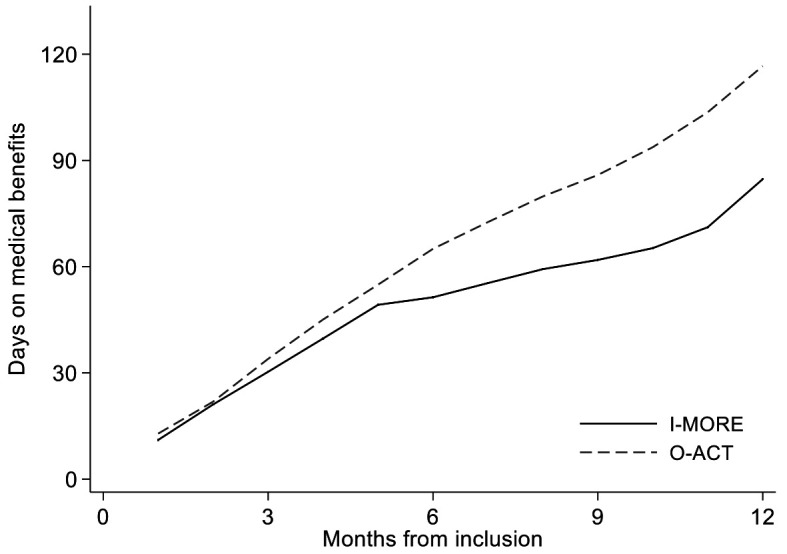
Sickness absence days during 12 months of follow up (cumulative median) for participants in inpatient multimodal occupational rehabilitation (I-MORE) and outpatient acceptance and commitment therapy (O-ACT).

The I-MORE participants had a median of 85 (IQR 33–149) SA days at 12-month follow-up, significantly less than the O-ACT group with 117 days (IQR 59–189; Mann-Whitney U-test; P=0.034). At 6 months follow-up, the median number of SA days was 51 (IQR 27–85) for I-MORE and 65 (IQR 42–97) O-ACT, respectively (Mann-Whitney U-test; P=0.114), see figure 2.

**Figure 3:**
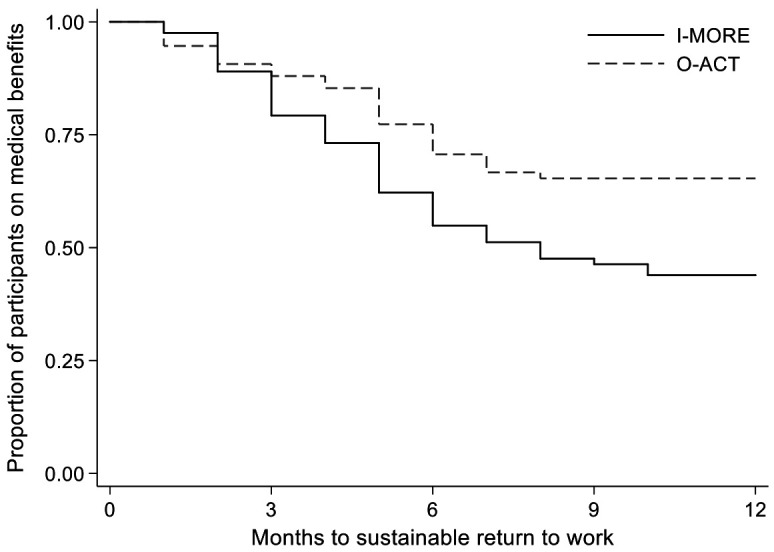
Time to sustainable return to work (Kaplan-Meier survival analysis) for participants in inpatient multimodal occupational rehabilitation (I-MORE) and outpatient acceptance and commitment therapy (O-ACT)

In total, 50 of the 86 participants in I-MORE and 31 of the 80 participants in O-ACT achieved sustainable return to work. Figure 3 shows the Kaplan-Meier plot. The difference between the programs was statistically significant (log rank test, P=0.009). The unadjusted return-to-work hazard ratio was 1.9 (95% CI 1.2–3.0), in favor of I-MORE and was unchanged after adjusting for age, gender, level of education, length and cause of sick leave (1.9; 95% CI 1.2–3.2).

The sensitivity analyses defining return to work as 2 and 3 months without receiving benefits showed similar hazard ratios (1.8 and 1.7) as the main analyses.

### Per protocol analysis

The median number of SA days during 12 months follow-up was 90 (IQR 33–170) versus 108 (IQR 58–156) days for I-MORE (N=69) and O-ACT (N=61), respectively (P=0.30). The respective sustainable return-to-work rates were 55% (N=38) and 43% (N=26) and the unadjusted hazard ratio was 1.4 (95% CI 0.85–2.44, P=0.17).

### Self-reported health and quality of life

There were no statistically significant differences between the programs in these secondary outcomes during 12 months of follow-up, except for a small difference in average pain in favor of O-ACT (estimated mean difference -0.95, 95% CI -1.7– -0.2 on a 0–10 numeric rating scale). Both groups improved anxiety, depression, and quality of life outcomes during follow up (table 3).

**Table 3:** Self-reported health outcomes. Numbers are estimates from unadjusted linear mixed models with random intercept and slope. I-MORE=inpatient multimodal occupational rehabilitation; O-ACT=outpatient acceptance and commitment therapy; CI=confidence interval.]

	Follow-up times	I-MORE (n=86)		O-ACT (n=80)		Effect estimates ^a^	

		Mean	95% CI	Mean	95% CI	Mean	95% CI
Quality of life ^b^ (0–1) ^c^	Start intervention	0.78	0.76–0.80	0.77	0.74–0.79		
	3 months	0.82	0.80–0.85	0.81	0.78–0.83		
	6 months	0.82	0.80–0.84	0.82	0.79–0.85		
	12 months	0.82	0.79–0.85	0.83	0.80–0.86	-0.02	-0.06–0.02
Depression ^b^ (0–21) ^d^	Baseline	5.7	4.9–6.6	6.6	5.7–7.5		
	Start intervention	5.9	5.0–6.8	7.1	6.1–8.0		
	End intervention	4.9	4.1–5.8	6.0	5.0–7.0		
	3 months	4.8	3.8–5.8	6.0	4.9–7.0		
	12 months	4.7	3.5–5.9	5.1	3.8–6.3	-0.72	-2.3–0.9
Anxiety ^b^ (0–21) ^d^	Baseline	7.4	6.5–8.2	8.6	7.7–9.5		
	Start intervention	7.7	6.8–8.5	8.4	7.4–9.3		
	End intervention	6.3	5.4–7.2	8.3	7.3–9.3		
	3 months	6.3	5.4–7.3	7.9	6.9–9.0		
	12 months	6.1	5.0–7.2	6.6	5.4–7.8	-0.22	-1.7–1.3
Average pain ^b^ (0–10) ^e^	Baseline	5.0	4.5–5.4	4.8	4.4–5.3		
	Start intervention	4.5	4.0–4.9	4.6	4.1–5.1		
	End intervention	4.1	3.7–4.6	4.5	4.0–5.0		
	3 months	4.5	4.0–5.0	4.2	3.7–4.8		
	12 months	4.7	4.1–5.3	3.9	3.2–4.5	-0.95	-1.7– -0.2
Strongest pain ^f^ (0–10) ^e^	Baseline	6.5	6.0–6.9	6.2	5.7–6.7		
	Start intervention	5.8	5.3–6.3	5.7	5.2–9.3		
	End intervention	5.7	5.2–6.3	5.6	5.0–6.2		
	3 months	5.9	5.3–6.5	5.8	5.2–6.5		
	12 months	5.8	5.1–6.6	5.0	4.2–5.8	-0.82	-1.9–0.3
Health complaints (0–87) ^g^	Start intervention	16	14–18	17	15–20		
	3 months	15	13–17	16	14–18		
	12 months	15	13–17	16	14–18	-0.35	-3.1–2.4

a Estimated mean differences from start of intervention, I-MORE minus O-ACT.

b Improvement for both interventions over time (P<0.05).

c Measured by 15D.

d Measured by the Hospital Anxiety and Depression Scale.

e Measured by numeric rating scale (pain last week).

f Improvement over time for O-ACT (P=0.01).

g Measured by the Subjective Health Complaints Inventory total score (modelled with random intercept only due to lack of convergence).

## Discussion

As hypothesized, I-MORE reduced SA more than O-ACT, and the time to sustainable return to work was shorter for I-MORE. Self-reported health outcomes (pain, distress and health-related quality of life) were largely similar between the groups during one year of follow up.

Our previous investigation of a shorter (8 days) inpatient program did not reduce SA compared to O-ACT (21). We are not aware of other studies that have examined the effect of a comprehensive inpatient occupational rehabilitation program comparable to our current study. In Norway, an intensive outpatient program consisting of six hours of daily activities for four weeks showed no overall effect on return to work compared to ordinary treatment in primary care (34). However, the same research group later reported that the individuals with the most complex problems returned to work faster when given the intensive rehabilitation program (35). Also, in a Norwegian study providing work-focused cognitive therapy combined with job support to individuals with common mental disorders, only the sub-group of individuals with the most complex problems and the longest SA benefitted from the intervention, and the effect on increased work participation was sustained after 4 years of follow up (36). Similar to the aforementioned studies (35, 36), the individuals in our study were long-term sickness absent (median 210 days in the preceding year).

Several factors could explain the superiority of I-MORE versus O-ACT impact on SA. As this study did not utilize a factorial design, it is not possible to ascribe the superiority of I-MORE to specific contrasts. The most notable differences between the programs were that I-MORE was inpatient, more intensive and multimodal – incorporating physical exercise and psychoeducational sessions. Living at the rehabilitation center for 3.5 weeks provided a break from daily life and gave more time for contemplation, discussion with peers, and integration of new coping strategies. The regulated schedule and a fixed wake-up time may have provided a frame for improved sleep and better coping with fatigue (37, 38). Psychoeducational sessions alone did not enhance return to work in a Danish study (39), but in synergy with other components of an inpatient multimodal intervention it might have contributed positively. We previously reported that a sub-sample of participants in I-MORE improved their cardiorespiratory fitness during the program, and increased further after a year (40). Still, we found little support that differences in self-reported health outcomes (table 3), or changes in expectancies about return to work (41), could explain the differences in SA between programs. This is in line with other studies observing that returning to work and improving health outcomes are not necessarily concurrent events (42, 43). Moreover, participants in O-ACT did not create a return-to-work plan, but an action plan in accordance with their most important values. This may also explain why I-MORE improved work outcomes compared with O-ACT.

Workplace involvement is considered a critical factor in effective return to work programs (6), but our results suggest that I-MORE interventions can be successful without this component. Another study from our group provided no evidence that adding a workplace intervention could further improve work participation outcomes (44). Finally, also considering our previous negative findings of a shorter inpatient program (21), our results support the current practice in Norway of 3–4 weeks of inpatient occupational rehabilitation.

A particular strength of this randomized study is the use of high-quality sick leave registry data, which assured complete data regarding SA and return to work. In contrast, less than half of the participants answered the questionnaires at the 12-month follow-up. Assuming missing at random, the mixed-model approach alleviates this problem by applying likelihood-based analyses using all available data (33). The number of missing questionnaires were fairly similar for the two groups at 6 and 12 months, but we cannot disregard the possibility of an attrition bias for the secondary outcomes. Blinding of participants and caregivers regarding allocation was not feasible. Primary researchers were not blinded in preparation of the dataset. However, one of the authors were blinded to allocation and performed a separate analysis of the primary outcome measures before commencing with further analyses and discussing the findings. Moreover, the employees at the Norwegian Labor and Welfare Service that prospectively register SA data were unaware of group allocation. Another particular strength of the study design was that the Norwegian Labor and Welfare Service invited participants among those fitting the eligibility criteria in the registry, eliminating referral bias and potentially increasing the external validity of the results. However, only 38% (of 3808 invited) responded, and only 271 underwent a full clinical multidisciplinary eligibility assessment (figure 1). Since we do not know how many of those not responding that would have fulfilled the eligibility criteria, we cannot rule out a “self-selection” bias, possibly limiting the generalizability of the results to situations with similar recruitment methods. Another issue is that participants had to be willing to leave their home for 3.5 weeks to participate in I-MORE. Moreover, the differences in SA diminished in the per protocol analysis. This could be explained by the different patterns of withdrawal in I-MORE (before start) and O-ACT (during the intervention). It is conceivable that individuals that were able to return to work when the intervention started, would opt for this rather than 3.5 weeks of inpatient rehabilitation. Conversely, weekly O-ACT could be combined with work, making it unnecessary to withdraw before the program started. In addition, individuals who were unable to participate once a week were probably those least able to work. A limitation of our study is that we have no information on how O-ACT would have compared to usual care. Another limitation is that no scoring of therapists’ adherence to or competence in ACT was done. However, the same peer-reviewed ACT trainer supervised clinicians in both interventions. In addition, a focus group interview study showed that all the relevant ACT processes of behavioral change were reflected in the I-MORE participants’ experiences (20).

Finally, since legislation, social security systems and occupational rehabilitation services differ extensively between countries; one should consider contextual factors before implementing this intervention, especially in parts of the world other than the Nordic countries.

### Concluding remarks

Among individuals on long-term SA due to musculoskeletal or common mental health disorders, I-MORE over 3.5 weeks reduced SA compared with 6 weekly sessions of O-ACT in the year after inclusion. Studies with longer follow-up and economic evaluations should be performed.

## References

[1] Vos T, Flaxman AD, Naghavi M, Lozano R, Michaud C, Ezzati M (2012). Years lived with disability (YLDs) for 1160 sequelae of 289 diseases and injuries 1990-2010:a systematic analysis for the Global Burden of Disease Study 2010. Lancet.

[2] OECD (2016). Health at a Glance:Europe 2016.

[3] Knudsen AK, Overland S, Hotopf M, Mykletun A (2012). Lost working years due to mental disorders:an analysis of the Norwegian disability pension registry. PLoS One.

[4] Murray CJ, Atkinson C, Bhalla K, Birbeck G, Burstein R, Chou D (2013). The state of US health 1990-2010:burden of diseases, injuries, and risk factors. Jama.

[5] Costa-Black KM, Feuerstein M, Loisel P, Loisel P, Anema JR (2013). Work Disability Models:Past and Present. Handbook of Work Disability:Prevention and Management.

[6] Cullen KL, Irvin E, Collie A, Clay F, Gensby U, Jennings PA (2018). Effectiveness of workplace interventions in return-to-work for musculoskeletal, pain-related and mental health conditions:an update of the evidence and messages for practitioners. J Occup Rehabil.

[7] Finnes A, Enebrink P, Ghaderi A, Dahl J, Nager A, Ost LG (2019). Psychological treatments for return to work in individuals on sickness absence due to common mental disorders or musculoskeletal disorders:a systematic review and meta-analysis of randomized-controlled trials. Int Arch Occup Environ Health.

[8] Andersen MF, Nielsen KM, Brinkmann S (2012). Meta-synthesis of qualitative research on return to work among employees with common mental disorders. Scand J Work Environ Health.

[9] MacEachen E, Clarke J, Franche RL, Irvin E (2006). Systematic review of the qualitative literature on return to work after injury. Scand J Work Environ Health.

[10] Reme SE, Tangen T, Moe T, Eriksen HR (2011). Prevalence of psychiatric disorders in sick listed chronic low back pain patients. Eur J Pain.

[11] Barnett K, Mercer SW, Norbury M, Watt G, Wyke S, Guthrie B (2012). Epidemiology of multimorbidity and implications for health care, research, and medical education:a cross-sectional study. Lancet.

[12] Hara KW, Borchgrevink PC, Jacobsen HB, Fimland MS, Rise MB, Gismervik S (2017). Transdiagnostic group-based occupational rehabilitation for participants with chronic pain, chronic fatigue and common mental disorders A feasibility study. Disabil Rehabil.

[13] Sylvain C, Durand MJ, Velasquez Sanchez A, Lessard N, Maillette P (2019). Development and Implementation of a Mental Health Work Rehabilitation Program:Results of a Developmental Evaluation. J Occup Rehabil.

[14] Dindo L, Van Liew JR, Arch JJ (2017). Acceptance and Commitment Therapy:A Transdiagnostic Behavioral Intervention for Mental Health and Medical Conditions. Neurotherapeutics.

[15] ATjak JGL, Davis ML, Morina N, Powers MB, Smits JA, Emmelkamp PM (2015). A meta-analysis of the efficacy of acceptance and commitment therapy for clinically relevant mental and physical health problems. Psychother Psychosom.

[16] Hayes SC, Villatte M, Levin M, Hildebrandt M (2011). Open, aware, and active:contextual approaches as an emerging trend in the behavioral and cognitive therapies. Annual Rev Clin Psych.

[17] Veehof MM, Trompetter HR, Bohlmeijer ET, Schreurs KM (2016). Acceptance- and mindfulness-based interventions for the treatment of chronic pain:a meta-analytic review. Cog Behav Ther.

[18] Dahl J, Wilson KG, Nilsson A (2004). Acceptance and commitment therapy and the treatment of persons at risk for long-term disability resulting from stress and pain symptoms:A preliminary randomized trial. Behav Ther.

[19] Rise MB, Gismervik SO, Johnsen R, Fimland MS (2015). Sick-listed persons'experiences with taking part in an in-patient occupational rehabilitation program based on Acceptance and Commitment Therapy:a qualitative focus group interview study. BMC Health Serv Res.

[20] Gismervik SO, Fimland MS, Fors EA, Johnsen R, Rise MB (2018;Aug). The acceptance and commitment therapy model in occupational rehabilitation of musculoskeletal and common mental disorders:a qualitative focus group study. Disabil Rehabil.

[21] Aasdahl L, Pape K, Vasseljen O, Johnsen R, Gismervik S, Halsteinli V (2018). Effect of Inpatient Multicomponent Occupational Rehabilitation Versus Less Comprehensive Outpatient Rehabilitation on Sickness Absence in Persons with Musculoskeletal- or Mental Health Disorders:A Randomized Clinical Trial. J Occup Rehabil.

[22] Aasdahl L, Pape K, Vasseljen O, Johnsen R, Gismervik S, Jensen C (2017). Effects of Inpatient Multicomponent Occupational Rehabilitation versus Less Comprehensive Outpatient Rehabilitation on Somatic and Mental Health:Secondary Outcomes of a Randomized Clinical Trial. J Occup Rehabil.

[23] Schulz KF, Altman DG, Moher D (2010). CONSORT 2010 Statement:updated guidelines for reporting parallel group randomised trials. BMC Med.

[24] Fimland MS, Vasseljen O, Gismervik S, Rise MB, Halsteinli V, Jacobsen HB (2014). Occupational rehabilitation programs for musculoskeletal pain and common mental health disorders:study protocol of a randomized controlled trial. BMC Pub Health.

[25] Cleeland CS, Ryan KM (1994). Pain assessment:global use of the Brief Pain Inventory. Ann Acad Med Singapore.

[26] Zigmond AS, Snaith RP (1983). The hospital anxiety and depression scale. Acta Psychiatr Scand.

[27] Eriksen HR, Ihlebaek C, Ursin H (1999). A scoring system for subjective health complaints (SHC). Scand J Pub Health.

[28] Vartiainen P, Heiskanen T, Sintonen H, Roine RP, Kalso E (2016). Health-related quality of life and burden of disease in chronic pain measured with the 15D instrument. Pain.

[29] Fimland MS, Vasseljen O, Gismervik S, Rise MB, Halsteinli V, Jacobsen HB (2014). Occupational rehabilitation programs for musculoskeletal pain and common mental health disorders:study protocol of a randomized controlled trial. BMC Public Health.

[30] Rosner B (2011). Fundamentals of biostatistics.

[31] Efron B (1977). The efficiency of Cox's likelihood function for censored data. Journal of the Am Stat Assoc.

[32] Schoenfeld D (1982). Partial residuals for the proportional hazards regression model. Biometrika.

[33] Tango T (2016). On the repeated measures designs and sample sizes for randomized controlled trials. Biostatistics.

[34] Haldorsen EMH, Kronholm K, Skouen JS, Ursin H (1998). Multimodal cognitive behavioral treatment of patients sicklisted for musculoskeletal pain - A randomized controlled study. Scand J Rheum.

[35] Haldorsen EMH, Grasdal AL, Skouen JS, Risa AE, Kronholm K, Ursin H (2002). Is there a right treatment for a particular patient group?Comparison of ordinary treatment, light multidisciplinary treatment, and extensive multidisciplinary treatment for long-term sick-listed employees with musculoskeletal pain. Pain.

[36] Overland S, Grasdal AL, Reme SE (2018). Long-term effects on income and sickness benefits after work-focused cognitive-behavioural therapy and individual job support:a pragmatic, multicentre, randomised controlled trial. Occup Environ Med.

[37] Kallestad H, Jacobsen HB, Landro NI, Borchgrevink PC, Stiles TC (2015). The role of insomnia in the treatment of chronic fatigue. J Psychosom Res.

[38] Qaseem A, Kansagara D, Forciea MA, Cooke M, Denberg TD (2016). Management of Chronic Insomnia Disorder in Adults:A Clinical Practice Guideline From the American College of Physicians Management of Chronic Insomnia Disorder in Adults. Ann Int Med.

[39] Pedersen P, Sogaard HJ, Labriola M, Nohr EA, Jensen C (2015). Effectiveness of psychoeducation in reducing sickness absence and improving mental health in individuals at risk of having a mental disorder:a randomised controlled trial. BMC Public Health.

[40] Nordstoga AL, Mork PJ, Steiro Fiml, and M (2018). Improved cardiorespiratory fitness after occupational rehabilitation in merged diagnostic groups. Ann Occup Environ Med.

[41] Aasdahl L, Pape K, Vasseljen O, Johnsen R, Fimland MS (2019). Improved Expectations About Length of Sick Leave During Occupational Rehabilitation Is Associated with Increased Work Participation. J Occup Rehabil.

[42] Ejeby K, Savitskij R, Ost LG, Ekbom A, Brandt L, Ramnero J (2014). Symptom reduction due to psychosocial interventions is not accompanied by a reduction in sick leave:results from a randomized controlled trial in primary care. Scand J Prim Health Care.

[43] Kamper SJ, Apeldoorn AT, Chiarotto A, Smeets RJ, Ostelo RW, Guzman J (2015). Multidisciplinary biopsychosocial rehabilitation for chronic low back pain:Cochrane systematic review and meta-analysis. BMJ.

[44] Skagseth M, Fimland MS, Rise MB, Johnsen R, Borchgrevink PC, Aasdahl L (2019). Effectiveness of adding a workplace intervention to an inpatient multimodal occupational rehabilitation program:A randomized clinical trial. Scand J Work Environ Health.

